# Supplementary material for: The role of cyclonic activity in tropical temperature-rainfall scaling
Source: Nat Commun. 2021 Nov 18;12:6732. doi: 10.1038/s41467-021-27111-z (PMC8602412; doi:10.1038/s41467-021-27111-z)
Supplement: Supplementary file 1 — Supplementary Information [file 41467_2021_27111_MOESM1_ESM.pdf]

The role of cyclonic activity in tropical temperature-rainfall scaling

Supplementary Information

Dominik Traxl[1,2*], Niklas Boers[2,3,4], Aljoscha Rheinwalt[1], Bodo Bookhagen[1]

[1]Institute of Earth and Environmental Science, University of Potsdam, Potsdam, Germany
[2]Potsdam Institute for Climate Impact Research, Potsdam, Germany
[3]Technical University of Munich, Germany; School of Engineering & Design, Earth System Modelling
[4]Global Systems Institute and Department of Mathematics, University of Exeter, Exeter, UK

[*]corresponding author: dominik.traxl@posteo.org.

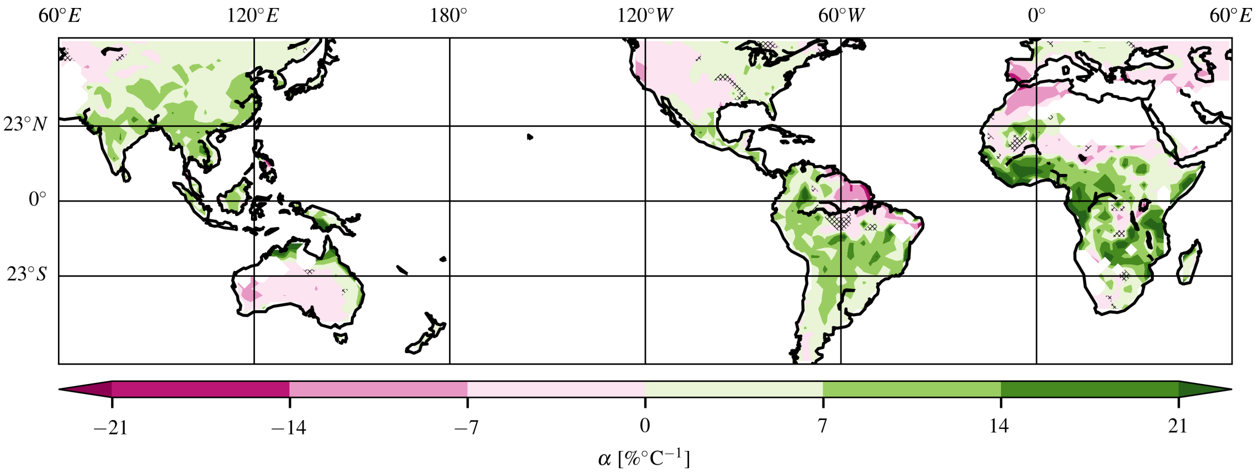

Supplementary Figure 1: Temperature-rainfall scaling over land masses. Spatial pattern of the relationship between temperatures T^r and rainfall intensities P^{90} over landmasses in July–August–September–October (JASO), in terms of fitted α-values (in [%°C^{-1}]). The colormap ranges from -3×CC (-21%°C^{-1}, pink) to +3×CC (+21%°C^{-1}, green). Pixels over water bodies, and pixels over landmasses with less than 100 data points per bin, are depicted as white. Pixels with a p-value larger than 5% are shaded with crossed black lines.

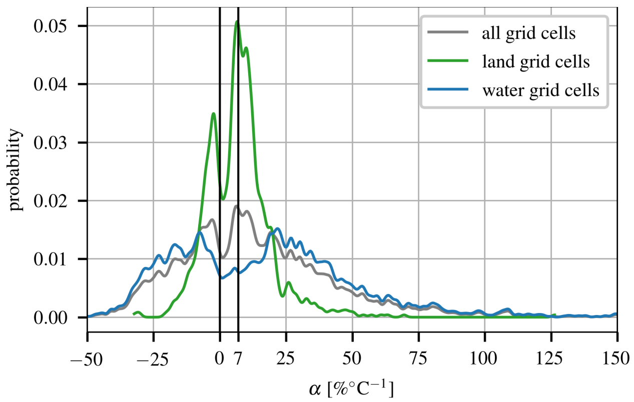

Supplementary Figure 2: Probability density functions of temperature-rainfall scaling factors over tropical oceans. Based on the per-pixel temperature-rainfall scaling factors (α-values) depicted in Fig. 1a and Supplementary Fig. 1, the probability density of α-values over tropical oceans (between 23°S and 23°N) for land, water and all grid cells is depicted.

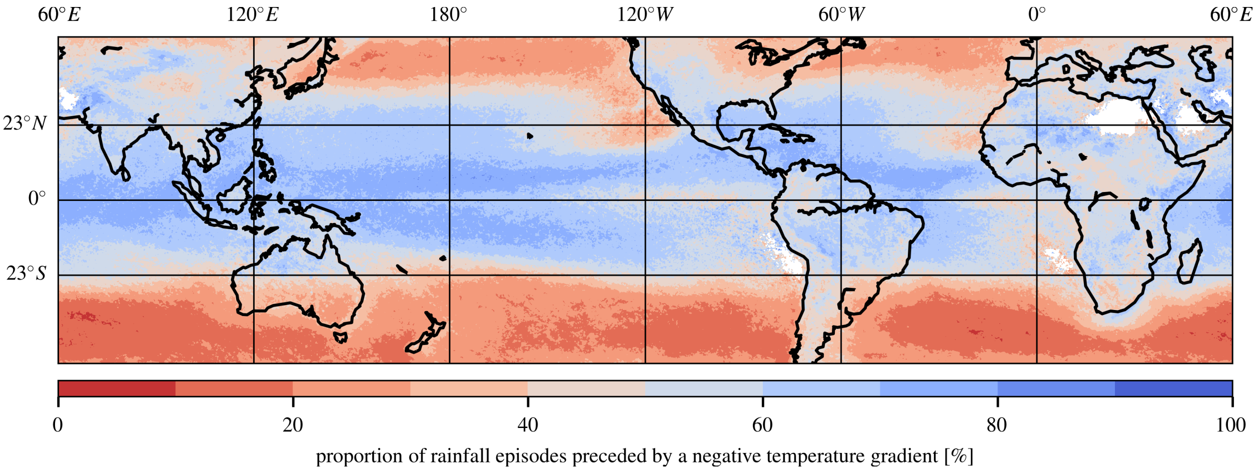

Supplementary Figure 3: Proportion of rainfall episodes preceded by a negative temporal temperature gradient. Spatial pattern of the proportion of rainfall episodes (per pixel, in July–August–September–October) that are preceded by a negative temporal temperature gradient.

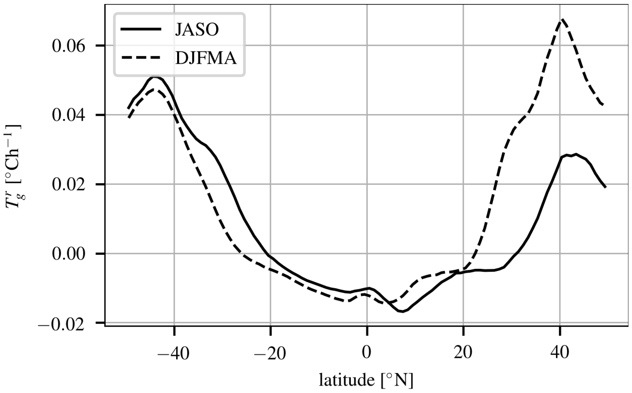

Supplementary Figure 4: Latitudinal profile of temporal temperature gradients preceding rainfall episodes. Rainfall episodes are binned according to their latitude, and for each bin the average temporal temperature gradient of the corresponding episodes is depicted (solid line for episodes occurring in July–August–September–October, dashed line for episodes in December–January–February–March–April).

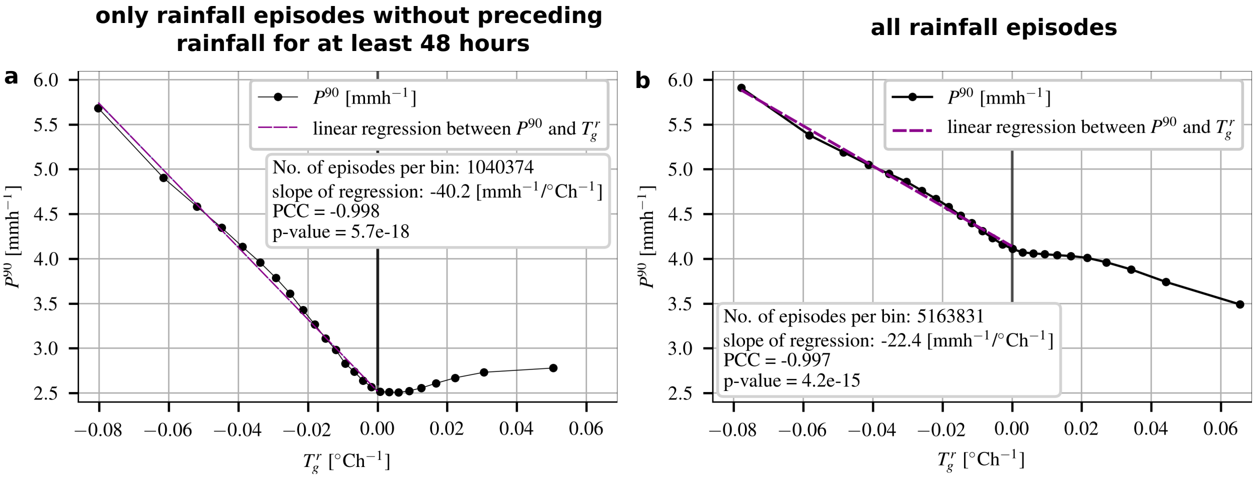

Supplementary Figure 5: Scaling between temporal temperature gradients and extreme rainfall intensities over northern tropical oceans for all episodes, and for episodes without preceding rainfall for at least 48 hours. **(a)** Same as Fig. 4a, illustrated again here for easier comparison with (b). **(b)** Same as (a), but considering all rainfall episodes, not only those without preceding rainfall for at least 48 hours.

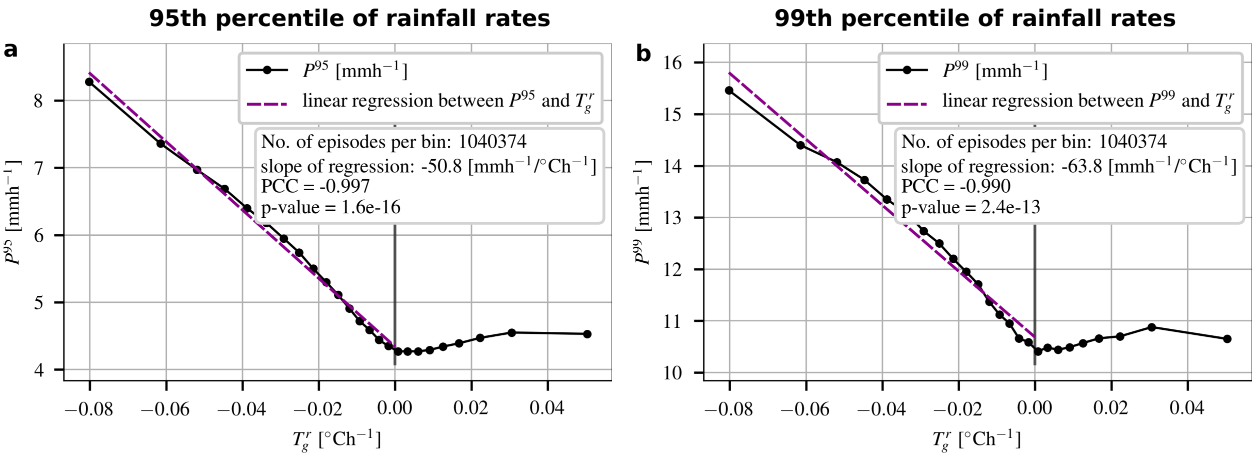

Supplementary Figure 6: Scaling between temporal temperature gradients and extreme rainfall intensities over northern tropical oceans considering the 95th and 99th percentile of intensities. **(a)** Same as Fig. 4a, but considering the 95th percentile of rainfall rates. **(b)** Same as Fig. 4a, but considering the 99th percentile of rainfall rates.

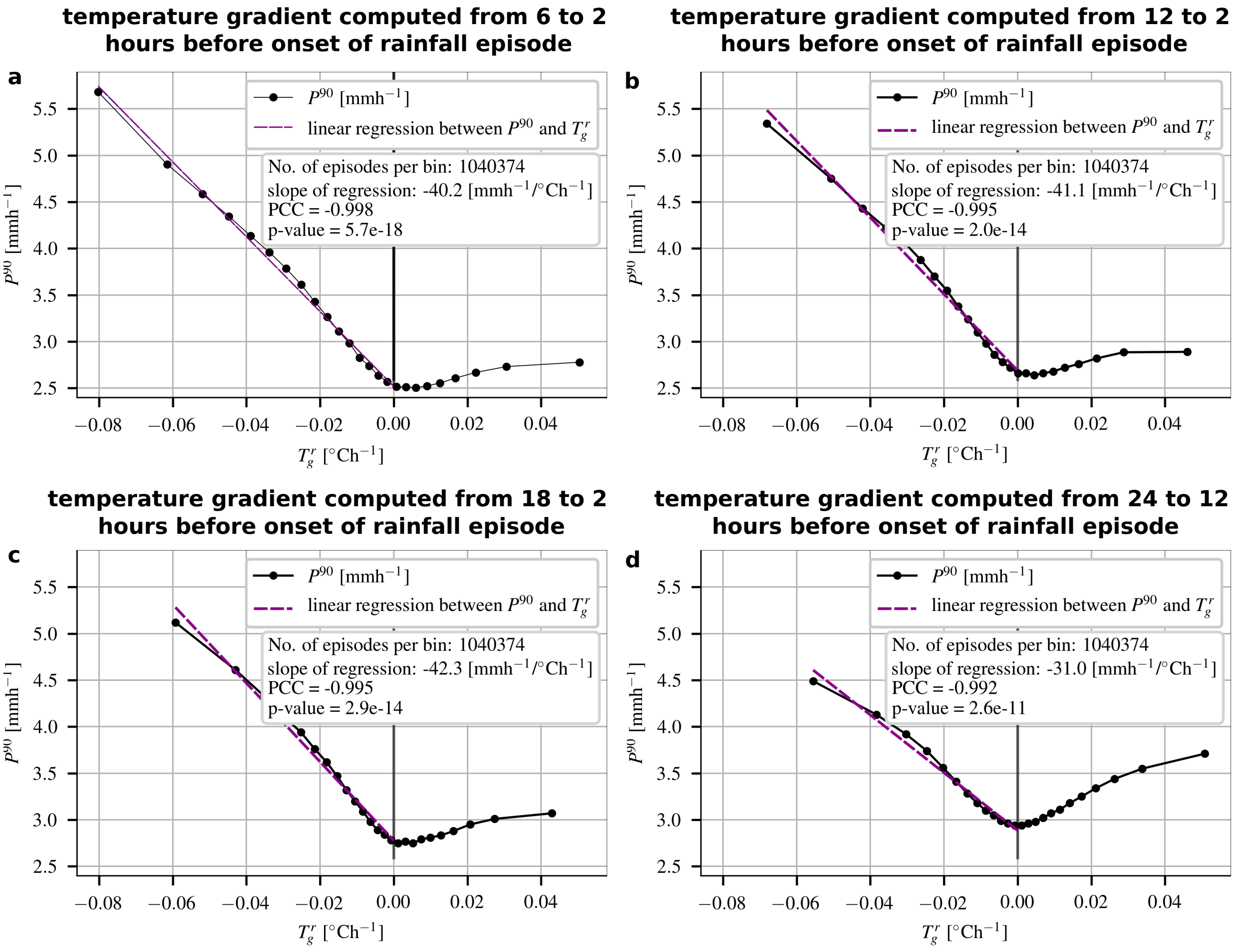

Supplementary Figure 7: Scaling between temporal temperature gradients and extreme rainfall intensities over northern tropical oceans considering various temporal windows to compute the pre-rainfall temperature gradient. **(a)** Same as Fig. 4a, illustrated again here for easier comparison with (b), (c) and (d). **(b)** Same as (a), but using the rolling 24-hour mean temperature from 12 to 2 hours before the onset of the episode to compute the temporal pre-rainfall temperature gradient. **(c)** using a time-window from 18 to 2 hours before the onset. **(d)** using a time-window from 24 to 12 hours before the onset.

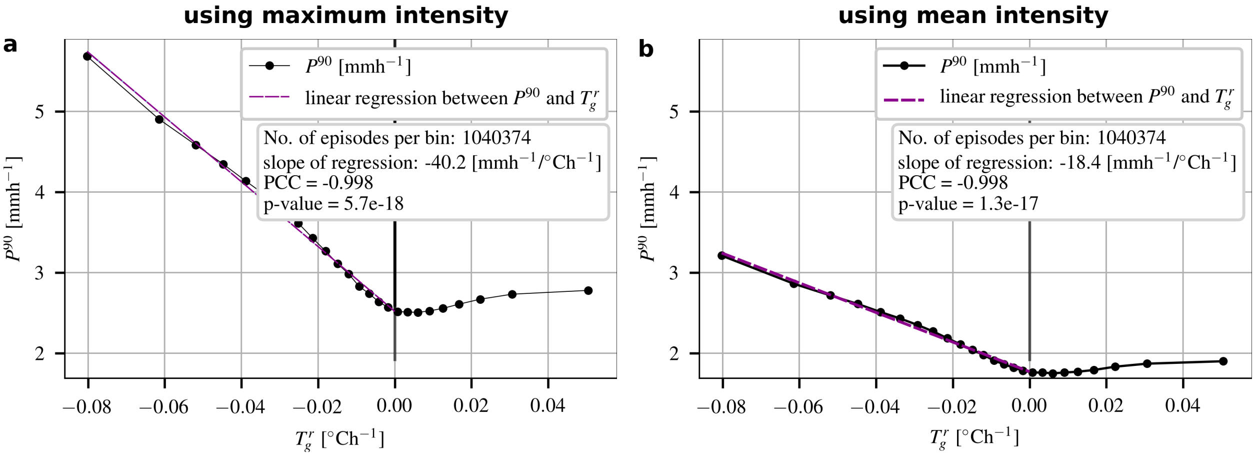

Supplementary Figure 8: Scaling between temporal temperature gradients and extreme rainfall intensities over northern tropical oceans using different definitions of the rainfall intensity of a rainfall episode. (a) Same as Fig. 4a, illustrated again here for easier comparison with (b) **(b)** Same as Fig. 4a, but using a different definition of the intensity of a episode: here, we use the mean intensity of all events the episode is comprised of, rather than the maximum intensity, to define the intensity of an episode.

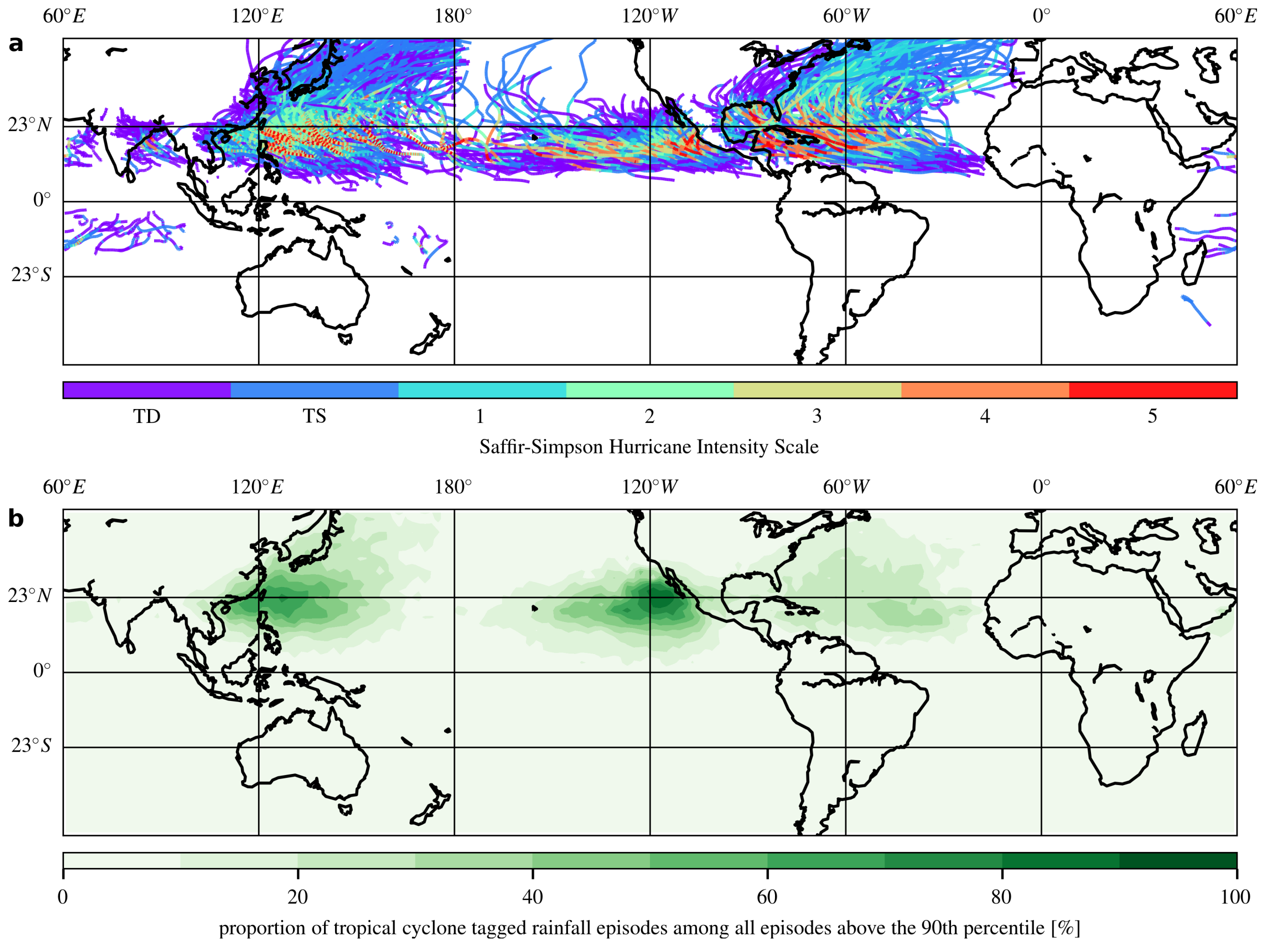

Supplementary Figure 9: Tropical cyclone tracks and contribution of rainfall associated with tropical cyclones to overall extreme rainfall. **(a)** Tracks and intensity of tropical cyclones (TCs) in July–August–September–October (JASO) as listed in the IBTrACS archive, recorded over the period from 1998 to 2018. Tracks are plotted on top of each other, whereby most intense storms are drawn last. **(b)** Per-pixel proportion of TC-tagged rainfall episodes among all episodes above the 90th percentile (in [%], only considering JASO).

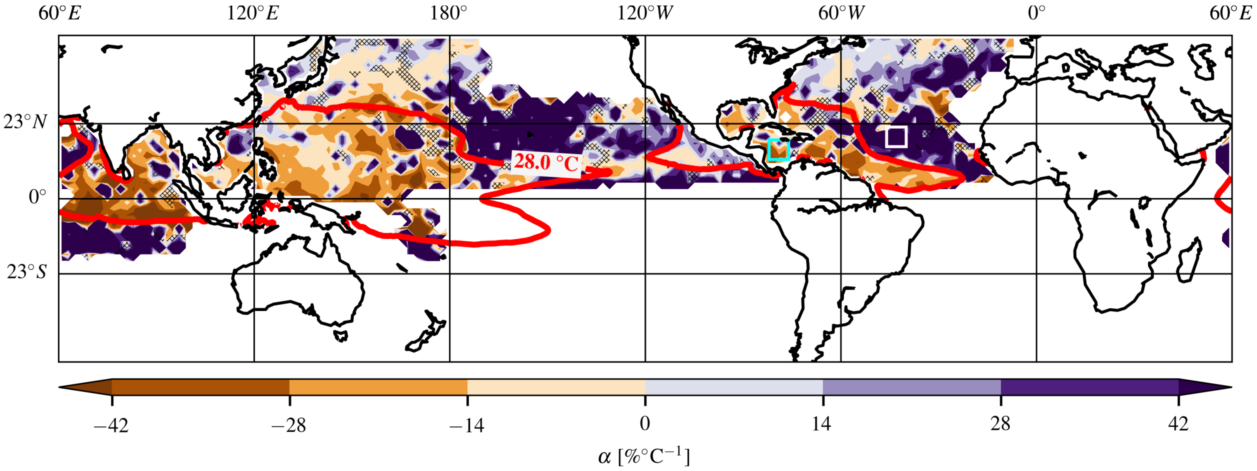

Supplementary Figure 10: Spatial distribution of temperature-rainfall scaling factors using only rainfall episodes associated with tropical cyclones. Spatial distribution of temperature-rainfall scaling factors (α-values, in [$\%°\mathrm{C}^{-1}$]) over water bodies in July–August–September–October. Only episodes that are associated with tropical cyclones listed in the IBTrACS archive are considered. Pixels over landmasses, and pixels over water bodies with less than 20 data points per bin, are depicted as white. Pixels with a p-value larger than 5% are shaded with crossed black lines.

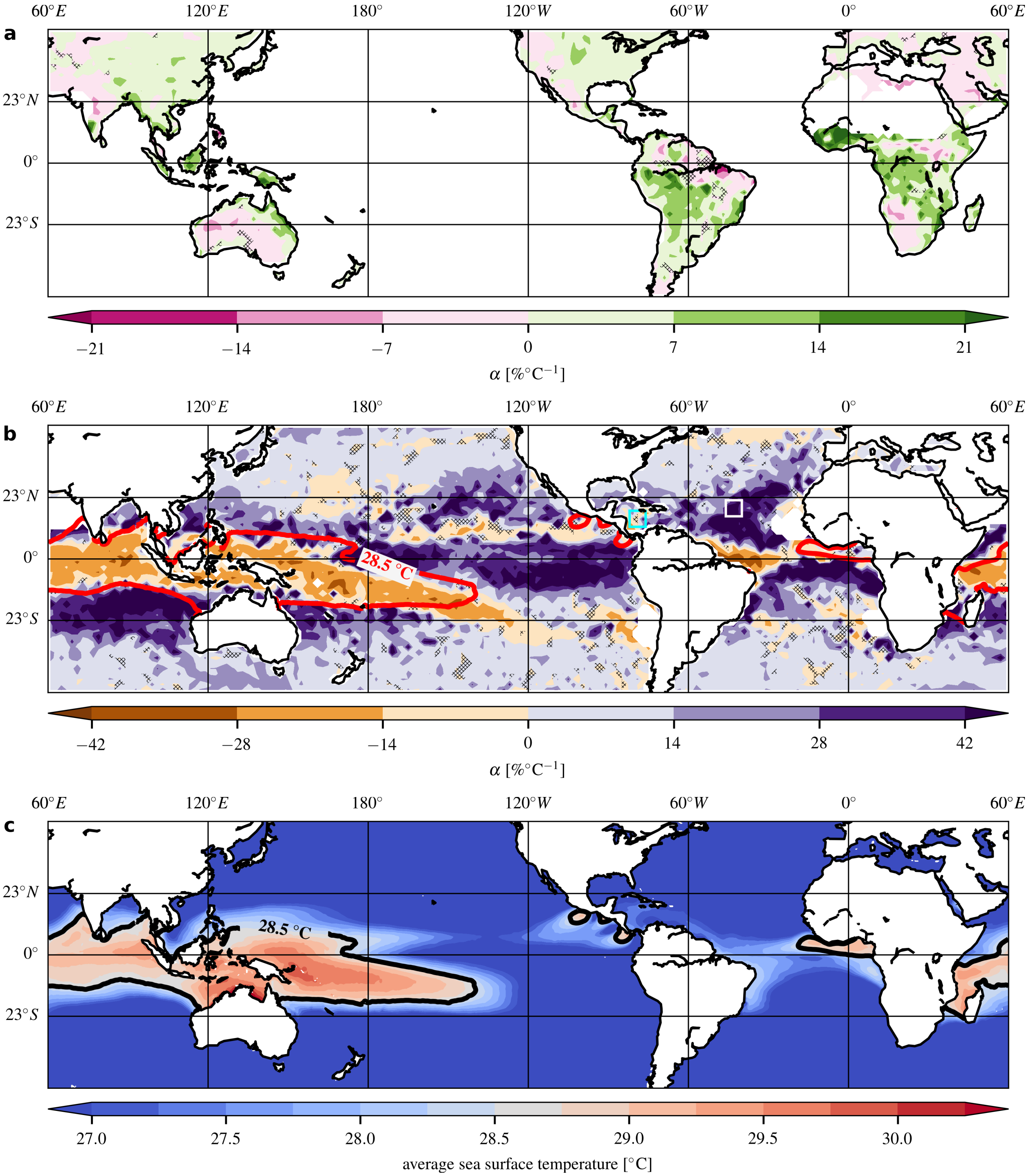

Supplementary Figure 11: Temperature-rainfall scaling and sea surface temperatures in December–January–February–March–April (DJFMA). Same as Supplementary Fig. 1 and Fig. 1, but for DJFMA.

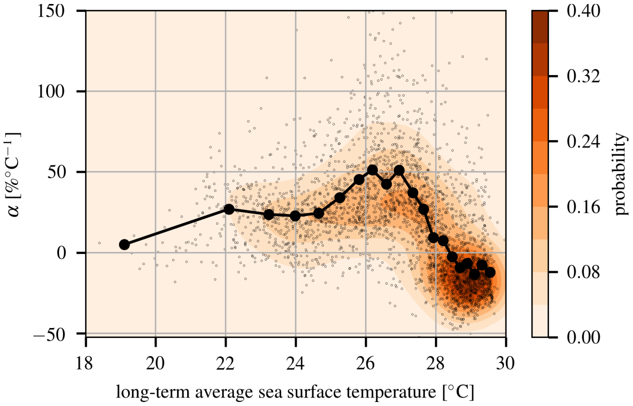

Supplementary Figure 12: Spatial correlation between average sea surface temperatures and temperature-rainfall scaling factors. The relationship between the temperature-rainfall scaling factors (α-values) and the long-term average sea surface temperatures (SSTs) over tropical oceans is shown. SST-α pairs are formed by spatial coincidence of the α and SST values depicted in Figs. 1a and b, respectively. Black empty circles depict the original SST-α pairs, the black line and filled circles indicate the average α-value for each SST bin. The colormap indicates the probability density based on a Gaussian kernel density estimation. Up to approximately 24°C, α-values are practically independent of SSTs. Between 24°C and 26°C, α-values increase with increasing SSTs. From 26°C upwards - approximately the temperature threshold for cyclogenesis - we obtain a strongly negative correlation between SSTs and α-values. Negative α-values are heavily concentrated above approximately 28°C, corresponding to the red contour line in Fig. 1a.

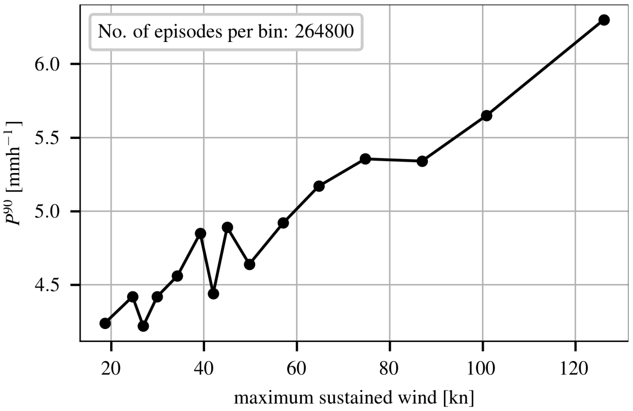

Supplementary Figure 13: Relationship between extreme rainfall intensity and the maximum sustained wind speed of tropical cyclones. Rainfall episodes (over northern tropical oceans in JASO) associated with tropical cyclones (TCs) as listed in the IBTrACS archive are binned according to the maximum sustained wind speed (in [kn]) of the TC they are associated with. For each bin, the 90th percentile of intensities (in [mmh^{-1}]) is shown.

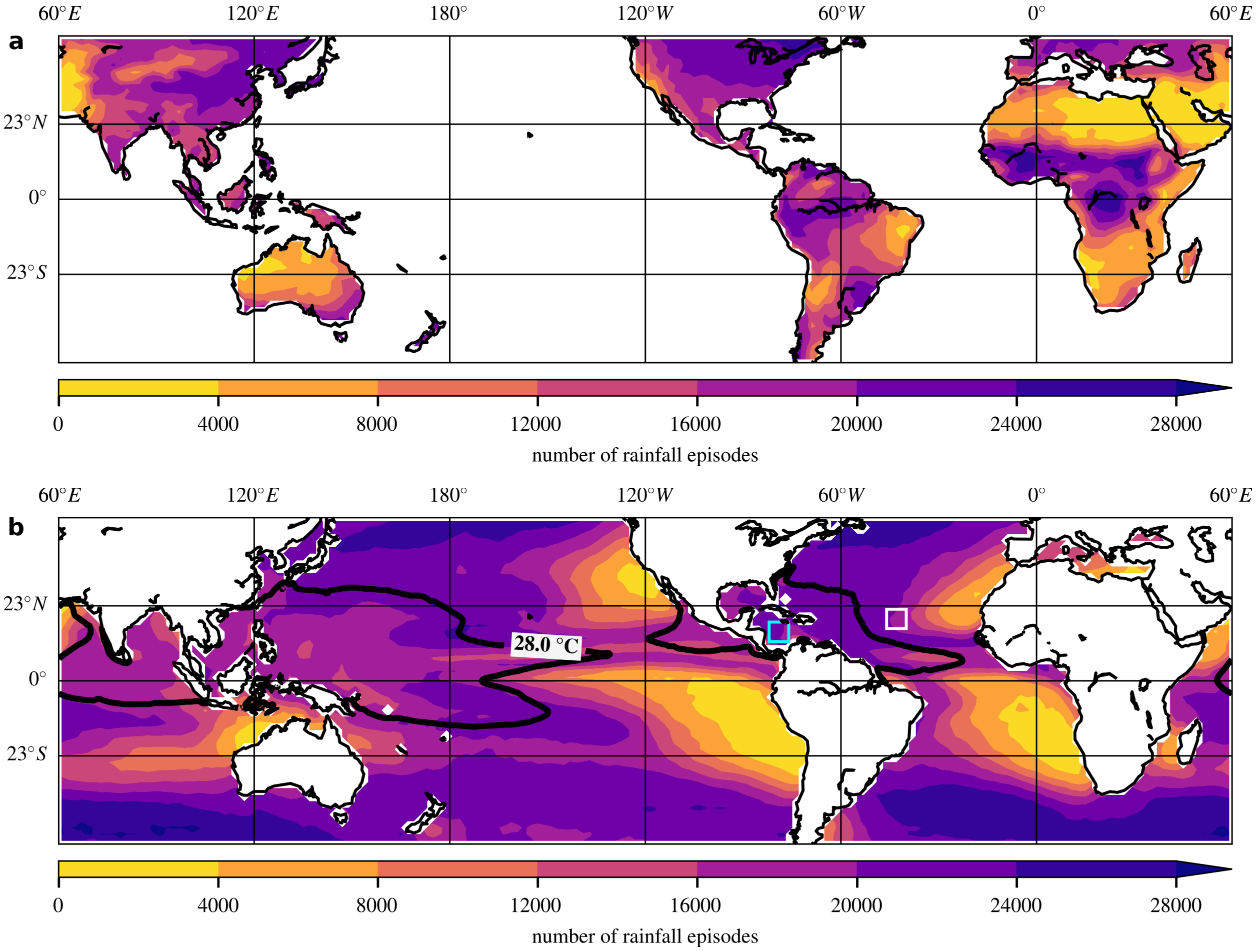

Supplementary Figure 14: Spatial distribution of the total number of rainfall episodes considered in the study. (a) The total number of episodes over landmasses in July–August–September–October for each grid location over the period from 1998 to 2018 is depicted. **(b)** Same as (a), but over water bodies. The black line illustrates the 28.0°C contour line as illustrated in Fig. 1b.

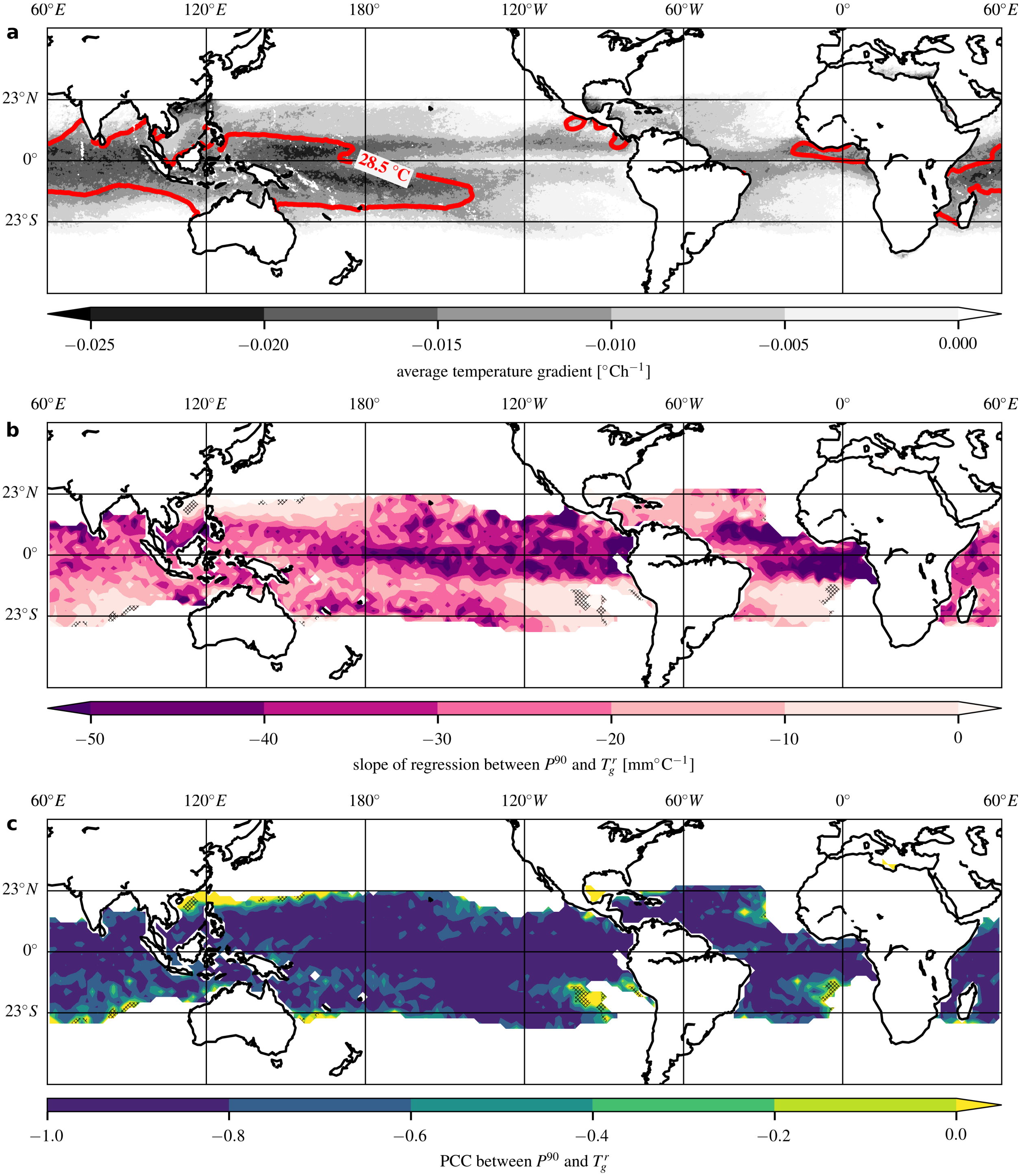

Supplementary Figure 15: Average temporal temperature gradients, scaling factors between temperature gradients and rainfall intensities and their corresponding linear correlation values. Same as Fig. 3, but for December–January–February–March–April.

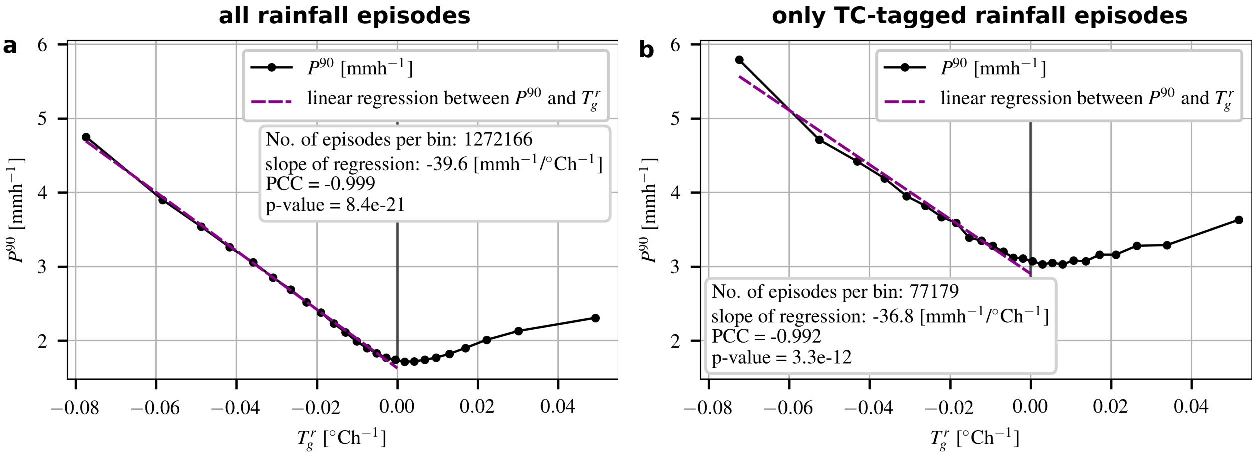

Supplementary Figure 16: Scaling between temporal temperature gradients and extreme rainfall intensities over northern tropical oceans. Same as Fig. 4, but for December–January–February–March–April.

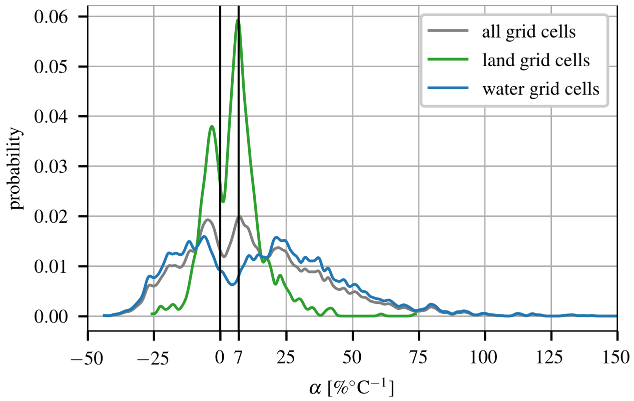

Supplementary Figure 17: Probability density functions of temperature-rainfall scaling factors over tropical oceans. Based on the per-pixel temperature-rainfall scaling factors (α-values) depicted in Supplementary Fig. 11a and b, the probability density of α-values over tropical oceans (between 23°S and 23°N) for land, water and all grid cells is depicted. Only December–January–February–March–April are considered.

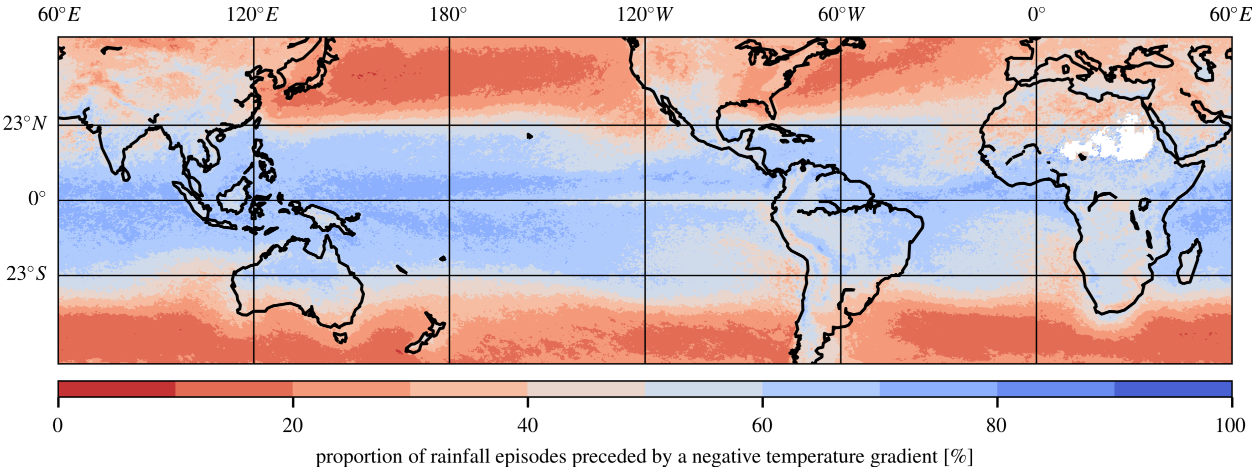

Supplementary Figure 18: Proportion of rainfall episodes preceded by a negative temporal temperature gradient. Same as Supplementary Fig. 3, but for December–January–February–March–April.

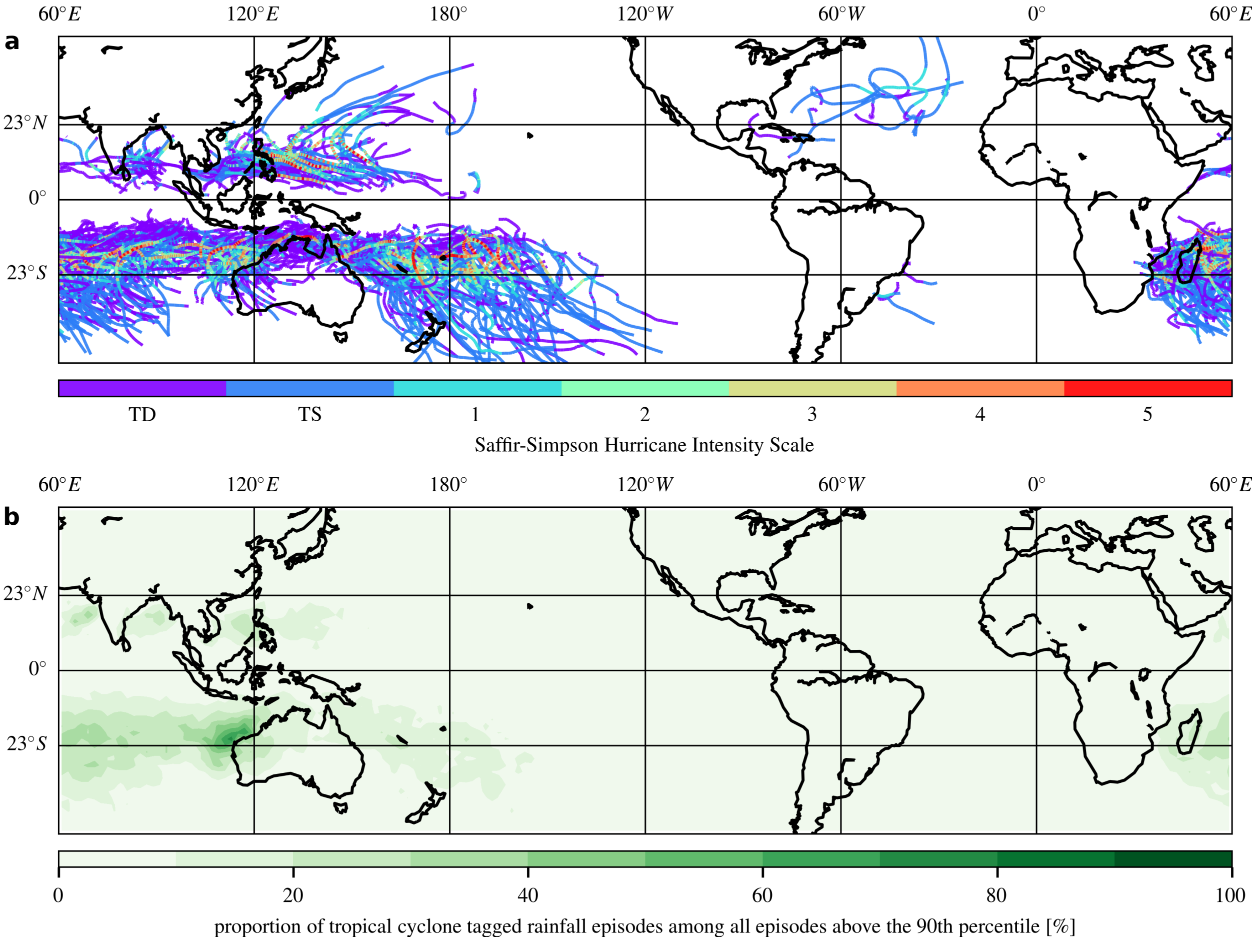

Supplementary Figure 19: Tropical cyclone tracks and contribution of rainfall associated with tropical cyclones to overall extreme rainfall. Same as Supplementary Fig. 9, but for December–January–February–March–April.

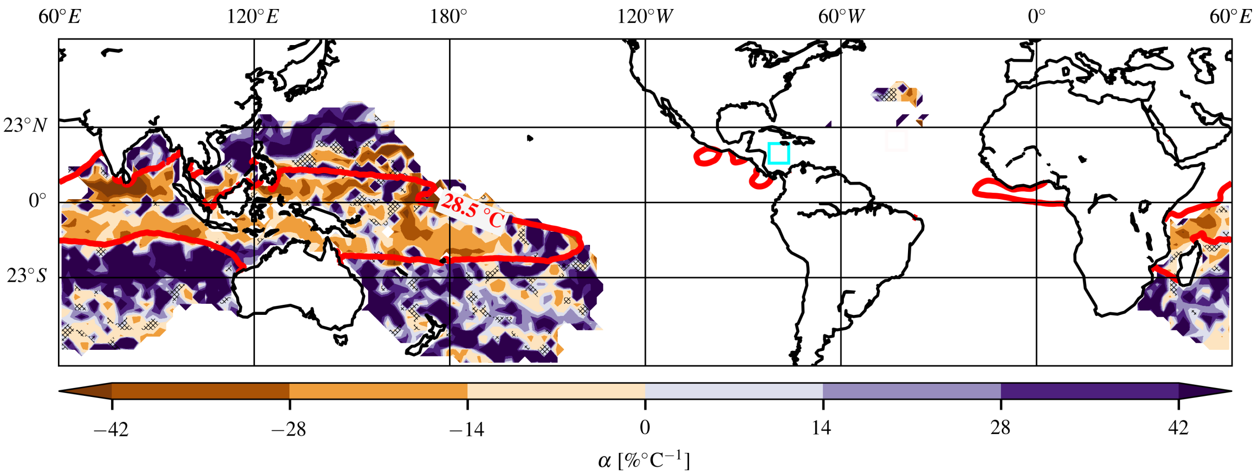

Supplementary Figure 20: Spatial distribution of temperature-rainfall scaling factors using only rainfall episodes associated with tropical cyclones. Same as Supplementary Fig. 10, but for December–January–February–March–April.

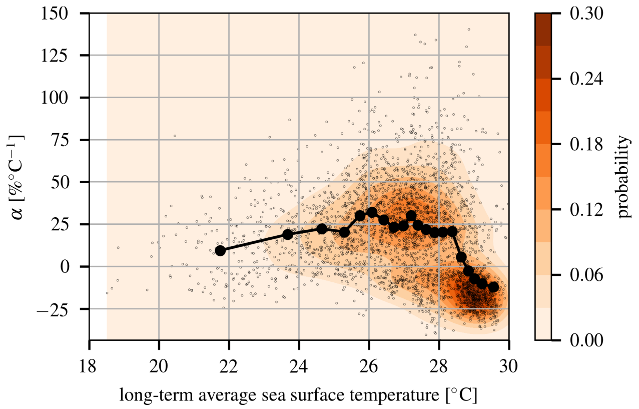

Supplementary Figure 21: Spatial correlation between average sea surface temperatures and temperature-rainfall scaling factors. Same as Supplementary Fig. 12, but for December–January–February–March–April.

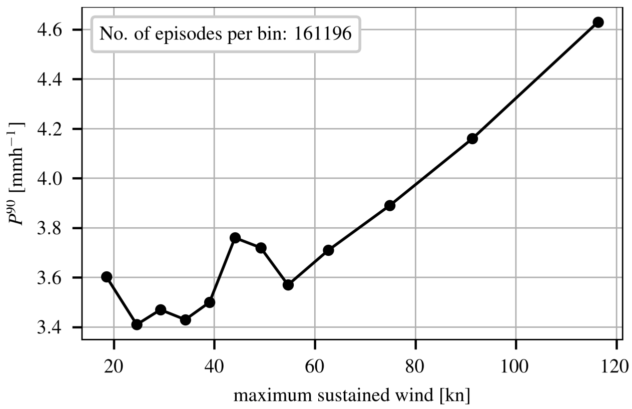

Supplementary Figure 22: Relationship between extreme rainfall intensity and the maximum sustained wind speed of tropical cyclones. Same as Supplementary Fig. 13, but for December–January–February–March–April.

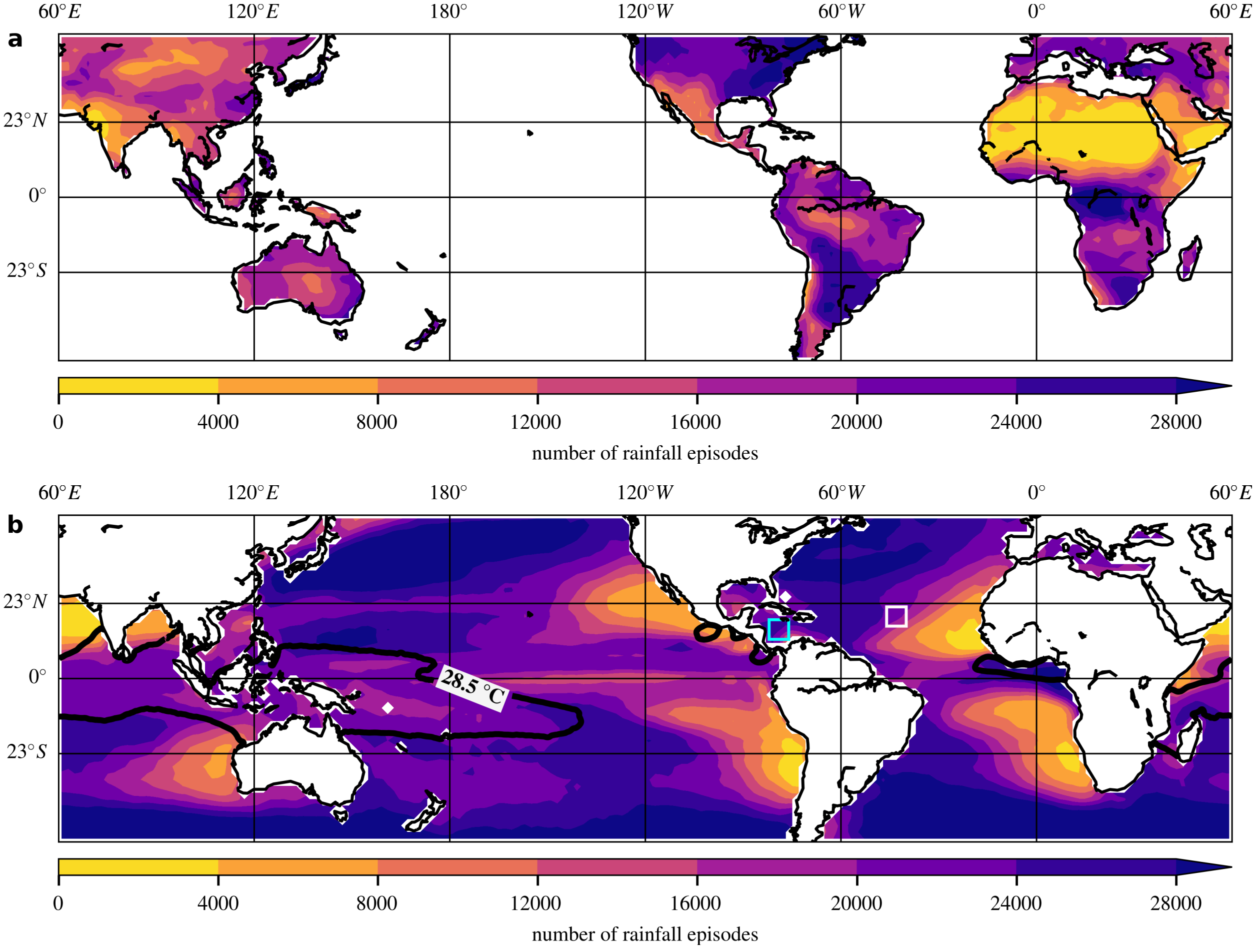

Supplementary Figure 23: Spatial distribution of the total number of rainfall episodes considered in the study. Same as Supplementary Fig. 14, but for December–January–February–March–April.
