# Supplementary material for: The role of cyclonic activity in tropical temperature-rainfall scaling
Source: Nat Commun. 2021 Nov 18;12:6732. doi: 10.1038/s41467-021-27111-z (PMC8602412; doi:10.1038/s41467-021-27111-z)
Supplement: Supplementary file 3 — Reporting Summary [file 41467_2021_27111_MOESM3_ESM.pdf]

nature portfolio

Corresponding author(s): Dominik Traxl

Last updated by author(s): Jul 5, 2021

Reporting Summary

Nature Portfolio wishes to improve the reproducibility of the work that we publish. This form provides structure for consistency and transparency in reporting. For further information on Nature Portfolio policies, see our Editorial Policies and the Editorial Policy Checklist.

Statistics

For all statistical analyses, confirm that the following items are present in the figure legend, table legend, main text, or Methods section.

n/a	Confirmed	
☐	☒	The exact sample size (*n*) for each experimental group/condition, given as a discrete number and unit of measurement
☒	☐	A statement on whether measurements were taken from distinct samples or whether the same sample was measured repeatedly
☐	☒	The statistical test(s) used AND whether they are one- or two-sided<br>*Only common tests should be described solely by name; describe more complex techniques in the Methods section.*
☐	☒	A description of all covariates tested
☒	☐	A description of any assumptions or corrections, such as tests of normality and adjustment for multiple comparisons
☒	☐	A full description of the statistical parameters including central tendency (e.g. means) or other basic estimates (e.g. regression coefficient) AND variation (e.g. standard deviation) or associated estimates of uncertainty (e.g. confidence intervals)
☐	☒	For null hypothesis testing, the test statistic (e.g. *F*, *t*, *r*) with confidence intervals, effect sizes, degrees of freedom and *P* value noted<br>*Give P values as exact values whenever suitable.*
☒	☐	For Bayesian analysis, information on the choice of priors and Markov chain Monte Carlo settings
☒	☐	For hierarchical and complex designs, identification of the appropriate level for tests and full reporting of outcomes
☐	☒	Estimates of effect sizes (e.g. Cohen's *d*, Pearson's *r*), indicating how they were calculated

Our web collection on statistics for biologists contains articles on many of the points above.

Software and code

Policy information about availability of computer code

Data collection	Only a browser and a ftp client was used to retrieve data.
Data analysis	All data analysis was performed using Python 3.9.6 and open source Python packages. All Python code to reproduce results and figures will be provided upon acceptance.

For manuscripts utilizing custom algorithms or software that are central to the research but not yet described in published literature, software must be made available to editors and reviewers. We strongly encourage code deposition in a community repository (e.g. GitHub). See the Nature Portfolio guidelines for submitting code & software for further information.

Data

Policy information about availability of data

All manuscripts must include a data availability statement. This statement should provide the following information, where applicable:
- Accession codes, unique identifiers, or web links for publicly available datasets
- A description of any restrictions on data availability
- For clinical datasets or third party data, please ensure that the statement adheres to our policy

All data used in this study is publicly available. For rainfall estimates, we used the Tropical Rainfall Measuring Mission (TRMM) 3B42 V7 dataset, available trough https://disc.gsfc.nasa.gov/datasets/TRMM_3B42_7/summary and downloaded from https://disc2.gesdisc.eosdis.nasa.gov/s4pa/TRMM_L3/TRMM_3B42.7/. For temperature estimates, we used the ERA5 reanalysis dataset, downloaded from https://cds.climate.copernicus.eu/cdsapp#!/dataset/reanalysis-era5-single-levels. For sea surface temperatures, we used the NOAA OI SST V2 High ResolutionDataset, available through https://psl.noaa.gov/data/gridded/data.noaa.oisst.v2.highres.html and downloaded from ftp://ftp.cdc.noaa.gov/Datasets/noaa.oisst.v2.highres/. For tropical cyclone tracks, we used the International Best Track Archive for ClimateStewardship (IBTRACS), available through https://www.ncdc.noaa.gov/ibtracs/and downloaded from https://www.ncei.noaa.gov/

data/international-best-track-archive-for-climate-stewardship-ibtracs/v04r00/access/netcdf/IBTrACS.ALL.v04r00.nc. To facilitate full reproduciblity, the Python code used to produce the results and figures of this study are available on github and zenodo: [a link will be inserted uponacceptance].

Field-specific reporting

Please select the one below that is the best fit for your research. If you are not sure, read the appropriate sections before making your selection.

☐ Life sciences ☐ Behavioural & social sciences ☒ Ecological, evolutionary & environmental sciences

For a reference copy of the document with all sections, see nature.com/documents/nr-reporting-summary-flat.pdf

Ecological, evolutionary & environmental sciences study design

All studies must disclose on these points even when the disclosure is negative.

Study description	We perform a global analysis of the relationship between temperature and extreme rainfall intensity. All data used in this study is publicly available. No experiments were performed, and the study did not involve any field work.
Research sample	All data used in this study is publicly available. For rainfall estimates, we used the Tropical Rainfall Measuring Mission (TRMM) 3B42 V7 dataset, available trough https://disc.gsfc.nasa.gov/datasets/TRMM_3B42_7/summary and downloaded from https://disc2.gesdisc.eosdis.nasa.gov/s4pa/TRMM_L3/TRMM_3B42.7/. For temperature estimates, we used the ERA5 reanalysis dataset, downloaded from https://cds.climate.copernicus.eu/cdsapp#!/dataset/reanalysis-era5-single-levels. For sea surface temperatures, we used the NOAA OI SST V2 High ResolutionDataset, available through https://psl.noaa.gov/data/gridded/data.noaa.oisst.v2.highres.html and downloaded from ftp://ftp.cdc.noaa.gov/Datasets/noaa.oisst.v2.highres/. For tropical cyclone tracks, we used the International Best Track Archive for ClimateStewardship (IBTRACS), available through https://www.ncdc.noaa.gov/ibtracs/and downloaded from https://www.ncei.noaa.gov/data/international-best-track-archive-for-climate-stewardship-ibtracs/v04r00/access/netcdf/IBTrACS.ALL.v04r00.nc.
Sampling strategy	Sample sizes in our study are given by the original data sources. All sample sizes in our statistical analyses are stated in either the main text or the figures. Sample sizes in our study range in their scale from hundreds to millions of data points.
Data collection	We downloaded all data through the websites/tools given by the data providers: We use the Tropical Rainfall Measuring Mission (TRMM) 3B42 V7 dataset, available trough https://disc.gsfc.nasa.gov/datasets/TRMM_3B42_7/summary and downloaded from https://disc2.gesdisc.eosdis.nasa.gov/s4pa/TRMM_L3/TRMM_3B42.7/. For temperature estimates, we used the ERA5 reanalysis dataset, downloaded from https://cds.climate.copernicus.eu/cdsapp#!/dataset/reanalysis-era5-single-levels. For sea surface temperatures, we used the NOAA OI SST V2 High ResolutionDataset, available through https://psl.noaa.gov/data/gridded/data.noaa.oisst.v2.highres.html and downloaded from ftp://ftp.cdc.noaa.gov/Datasets/noaa.oisst.v2.highres/. For tropical cyclone tracks, we used the International Best Track Archive for ClimateStewardship (IBTRACS), available through https://www.ncdc.noaa.gov/ibtracs/and downloaded from https://www.ncei.noaa.gov/data/international-best-track-archive-for-climate-stewardship-ibtracs/v04r00/access/netcdf/IBTrACS.ALL.v04r00.nc.
Timing and spatial scale	No data collection of our own was performed. Using only publicly available data, the analysis is performed on a (nearly) global scale (from 50°S to 50°N), from the year 1998 to 2018. Rainfall, temperature and sea surface temperatures are gridded on a spatial resolution of 0.25°. The temporal resolution of rainfall estimates is 3-hourly, of temperature estimates 1-hourly and of sea surface temperatures daily.
Data exclusions	No data were excluded from the analyses.
Reproducibility	No experiments were conducted. Reproducibility of our results is facilitated by providing all code used to generate our results and figures.
Randomization	We performed no (random) subsetting of the original data used for this study.
Blinding	Blinding was not relevant to our study, since we did not perform any experiments or field work.

Did the study involve field work? ☐ Yes ☒ No

Reporting for specific materials, systems and methods

We require information from authors about some types of materials, experimental systems and methods used in many studies. Here, indicate whether each material, system or method listed is relevant to your study. If you are not sure if a list item applies to your research, read the appropriate section before selecting a response.

Materials & experimental systems

n/a	Involved in the study
☒	☐ Antibodies
☒	☐ Eukaryotic cell lines
☒	☐ Palaeontology and archaeology
☒	☐ Animals and other organisms
☒	☐ Human research participants
☒	☐ Clinical data
☒	☐ Dual use research of concern

Methods

n/a	Involved in the study
☒	☐ ChIP-seq
☒	☐ Flow cytometry
☒	☐ MRI-based neuroimaging
